# Mantophasmatodea from the Richtersveld in South Africa with description of two new genera and species

**DOI:** 10.3897/zookeys.746.14885

**Published:** 2018-03-27

**Authors:** Benjamin Wipfler, Tobias Theska, Reinhard Predel

**Affiliations:** 1 Entomology Group, Institut für Zoologie und Evolutionsforschung, Friedrich-Schiller-University Jena, Erbertstr. 1, 07743 Jena, Germany; 2 Institut für Zoologie, Universität zu Köln, Zülpicher Str. 47b, 50674, Köln, Germany

**Keywords:** heelwalkers, Polyneoptera, lower neoptera, *Kuboesphasma*, *Minutophasma*, taxonomy, South Africa

## Abstract

Two new species and two new genera (*Kuboesphasma*, *Minutophasma*) of Mantophasmatodea that occur in the Richtersveld region of South Africa are described. *Kuboesphasma compactum* **gen. n., sp. n.** was found only in a small area near the village of Kuboes, while *Minutophasma richtersveldense* **gen. n., sp. n.** apparently inhabits a larger area in the Richtersveld. With these two new species, a total of four different mantophasmatodeans are known to live in this area. This is a remarkable exception to the remaining representatives of this order, where even a common occurrence of only two species is rare. We discuss this sympatry in the context of the phylogeny of the group. Additionally, we provide a map of the known distributions and a table with the most important taxonomic features of the mantophasmatodeans in the Richtersveld.

## Introduction

Mantophasmatodea were newly described in 2002 (Klass et al. 2002), which makes it by far the youngest of all insect orders. Since then, a remarkable amount of research was done on the group. Various studies presented detailed information about the morphology of almost all parts of the body (e.g., Dallai et al. 2003; Klass et al. 2003; Baum et al. 2007; Buder and Klass 2013; Wipfler et al. 2015), behavior (e.g., Eberhard and Eberhard 2013; Roth et al. 2014), distribution and biogeography (e.g., Predel et al. 2012; Proches 2014), physiology (e.g., Chown et al. 2006; Eberhard et al. 2010), ecology (e.g., Eberhard and Picker 2008; Roth et al. 2014) and intraordinal phylogeny (e.g., Damgaard et al. 2008; Predel et al. 2012). It was settled without doubt that Grylloblattodea, a very small insect order with a preference for cold habitats in western North America and northern East Asia, is the sistergroup of Mantophasmatodea (Misof et al. 2014; Wipfler et al. 2014). This accumulated research makes Mantophasmatodea one of the best studied insect orders today.

So far 18 species of Mantophasmatodea have been described (Klass et al. 2002, Klass et al. 2003, Zompro et al. 2003, Zompro and Adis 2006, Eberhard et al. 2011, Wipfler et al. 2012). These descriptions encompass 9 species from South Africa, all belonging to a monophyletic group (Austrophasmatidae) that inhabits the winter rainfall region of the Western and Northern Cape. The monotypic genera *Praedatophasma*, *Tyrannophasma*, *Pachyphasma*, and *Striatophasma* can be found in Namibia. The same applies to several representatives of *Mantophasma* and *Sclerophasma*, whose statuses as separate species are poorly resolved (Roth et al. 2014). Members of the genus *Tanzaniophasma* inhabit East Africa, with known populations in South Tanzania (Klass et al. 2002), Malawi (Roth et al. 2014), and Mozambique (Predel, unpublished data). Recent phylogenetic analyses of all known populations of Mantophasmatodea (Predel, unpublished data) revealed two additional taxa of Mantophasmatodea in the Richtersveld. This arid region is located in the far northwest of Southern Africa and thus within the summer- and winter-rain transition zone. It represents the only recognized arid biodiversity hotspot in the world (Myers et al. 2000). Together with *Praedatophasma maraisi* and *Namaquaphasma ookiepense*, which are known to reach their distribution boundaries in this area, these taxa increase the number of species in that relatively small region to four. This is exceptional for Mantophasmatodea, particularly since the different species are not closely related.

Here we describe the two new genera and two new species of Mantophasmatodea from the Richtersveld and present detailed information on how to distinguish the four sympatric species in this area.

## Material and methods

The used terminology follows Beutel et al. (2014) and for abdominal structures Klass et al. (2003). If not stated otherwise, the coloration refers to living specimens. Species descriptions are based on a designated holotype, but all available specimens were taken into account in order to assess the intraspecific variation.

The information for the specimens is given in a standard manner, i.e., locality, geographic coordinates, elevation, date of collection (month indicated in lower case Roman numerals), habitat information, collector, depository, and preparation. Female (♀) and male (♂) symbols indicate the sex.

In accordance with Zompro et al. (2003) and Wipfler et al. (2011), we took the following measurements: total length, length of pronotum, width of pronotum, length of mesonotum, width of mesonotum, length of metanotum, width of metanotum, distance between ventral edge of clypeus and dorsal edge of frontal tubercle (head height of Zompro et al. 2003), width of the head, head width over eyes, width between eyes, length of eye and width of eye. Additionally, we measured the distance between the ventral edge of the labrum and the dorsal-most point of the head capsule in frontal view (total heights of the head). Specimens were examined under a Zeiss Stemi SV11 with a calibrated ocular micrometer. Images of the habitats were taken with a Nikon 3300 camera.

A male (holotype) and a female paratype of each newly described species was critical point dried and subsequently glued to a needle. Then we photographed them with a Keyence VHX-2000 digital microscope. Parts of each species are illustrated in the standard views of dorsal, lateral and ventral. The head is additionally depicted in frontal view (frons being vertically) and the terminalia in caudal view. Subsequent images were edited with Adobe Photoshop and Illustrator (CS6).

The specimens referred below along with the abbreviations used in the text will be deposited in the following collections: SAMC – Iziko South African Museum, Cape Town, South Africa; ZFMK – Zoologisches Forschungsinstitut und Museum Alexander Koenig, Bonn, Germany; ZMBN – University Museum, University of Bergen, Bergen, Norway.

## Taxonomy

### Kuboesphasma gen. n.

Taxon classificationAnimaliaNotopteraMantophasmatidae

http://zoobank.org/E36A7CC9-55C1-4428-9C64-BBD5EC1D9026

#### Description and diagnosis.

**Figure 1.**
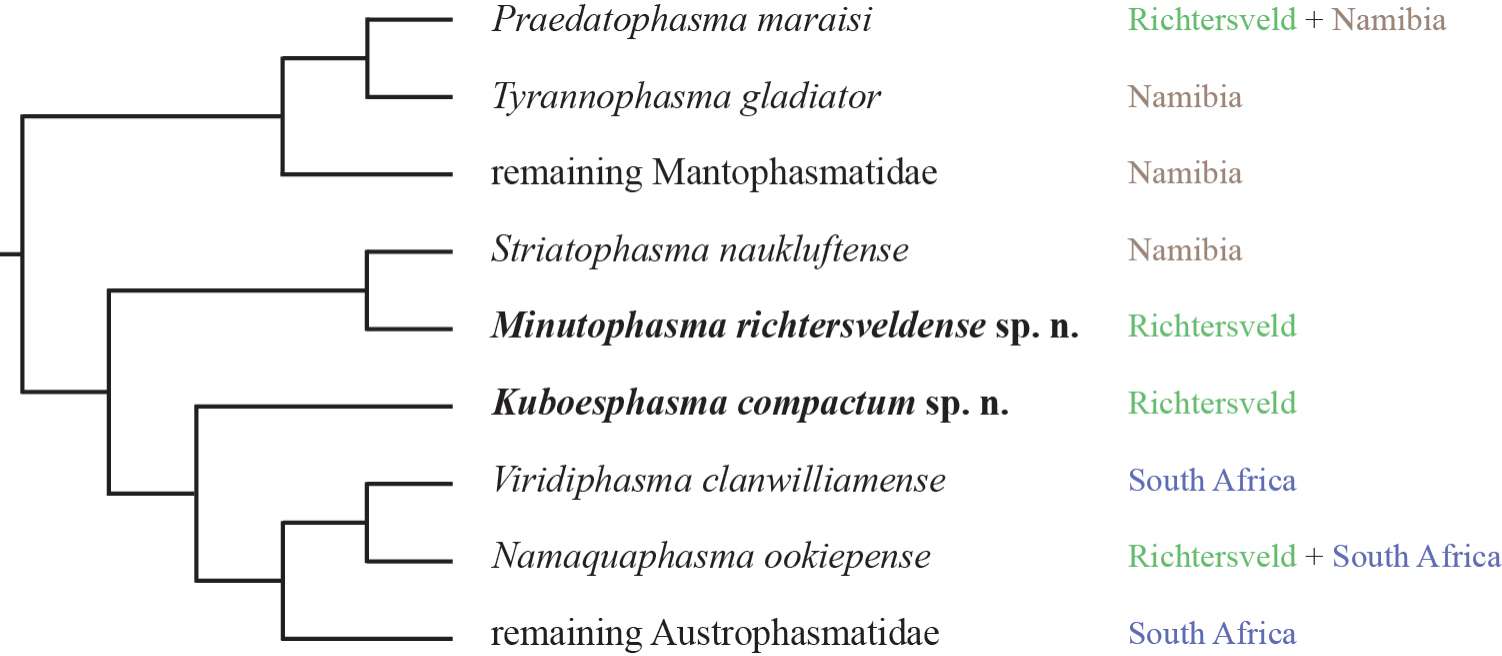
Simplified tree (midpoint-rooted) from a Bayesian phylogenetic analysis of peptide hormone sequences of southern African Mantophasmatodea (adapted from Predel et al., unpublished).

*Kuboesphasma* gen. n. is placed as sistergroup to a clade (*Viridiphasma* + *Namaquaphasma*) + the remaining Austrophasmatidae based on peptide hormone sequences (Predel, unpublished data; Fig. 1).

*Kuboesphasma* can be distinguished from other mantophasmatodeans, except *Minutophasma*, by the washed-out and indistinct butterfly-shaped spot on the frons: in the South African Austrophasmatidae sensu Klass et al. (2003) except *Viridiphasma clanwilliamense* this spot is clear and dark while *V. clanwilliamense* and the Namibian *Striatophasma*, *Pachyphasma* and Mantophasmatidae sensu Klass et al. (2003) lack it completely. Additionally, all green Austrophasmatidae and *Striatophasma* have a dark dorsal median stripe in males; this median stripe is indistinct in males of *Kuboesphasma*. *Kuboesphasma* can be distinguished from *Minutophasma* by the larger body size (males over 12 mm and females over 13 mm), the absence of the dark dorsal median stripe in males and the absence of black ventrolateral spot on the scapus. On the protibia, the males of *Kuboesphasma* have nine or more spikes per row (while *Minutophasma* has eight or less). Additionally, the head capsule is distinctly broader on the level of the compound eyes than on the level of the genae in *Minutophasma*, while they are equal in *Kuboesphasma*. Males and females of *Praedatophasma maraisi* are much larger (more than 21 mm) than those of *Kuboesphasma*, grey brown and have distinct spines along the thorax. In contrast to *Namaquaphasma ookiepense*, *Kuboesphasma* has no dark spots on the scapus, no distal dorsal projection on the cercus and an indistinct butterfly shaped spot on the frons. From *Striatophasma naukluftense* the species can be additionally separated by the rounded posterior margin of sternit VIII (pointed in *Striatophasma*) and the equal length of tergum VIII and IX (IX much longer in *Striatophasma*).

#### Type species.

*Kuboesphasma compactum*

#### Other included species.

None thus far.

#### Etymology.

The generic group name *Kuboesphasma* is a composition from the type locality, Kuboes, which is a center of the Nama community in the Richtersveld region and the ending -phasma which is commonly used to term mantophasmatodeans. The gender is neuter.

### Kuboesphasma compactum sp. n.

Taxon classificationAnimaliaNotopteraMantophasmatidae

http://zoobank.org/759D3A07-B689-4065-B602-DAB78E0E8AAC

Figs 2 , 3 , 4 , 5 , 6 , 7 , 8 , 9

#### Holotype.

Male. SOUTH AFRICA: Kuboes, S28°26'25", E16°59'44", 18.viii.2012, 250 m, R. Predel, specimen in ethanol. Table 1 provides an overview of the type material including the collections where it is deposited.

#### Paratypes.

SOUTH AFRICA: Richtersveld S28°26'25", E16°59'44", 18.viii.2012: four males and two females, specimens in ethanol. Table 1 provides an overview of the type material including the collections where it is deposited.

**Figure 2.**
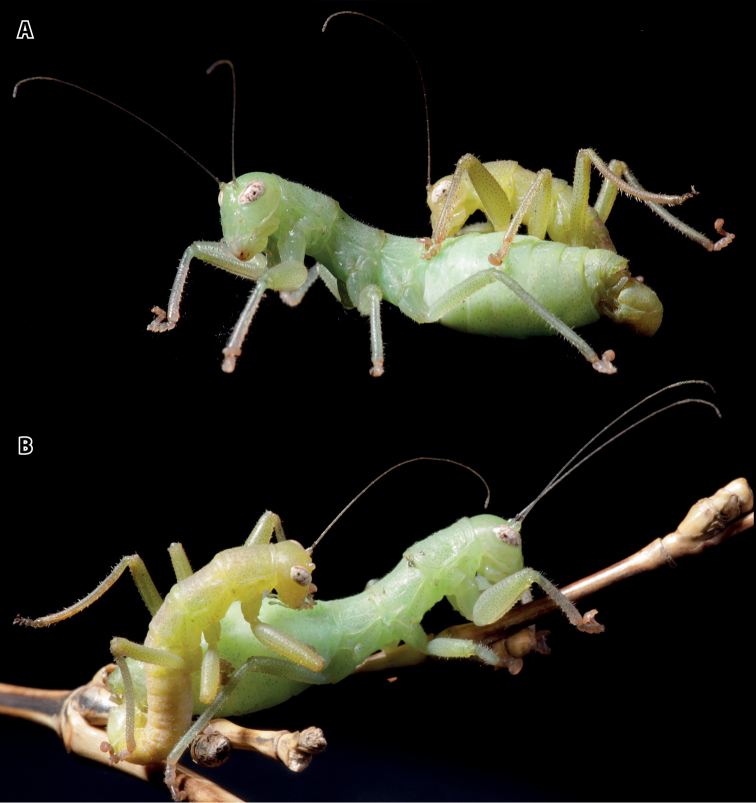
Habitus photographies of *Kuboesphasma compactum* sp. n.; copula with smaller ♂ on top of ♀; **A** lateral view from left side **B** dorso-lateral view from right side.

**Figure 3.**
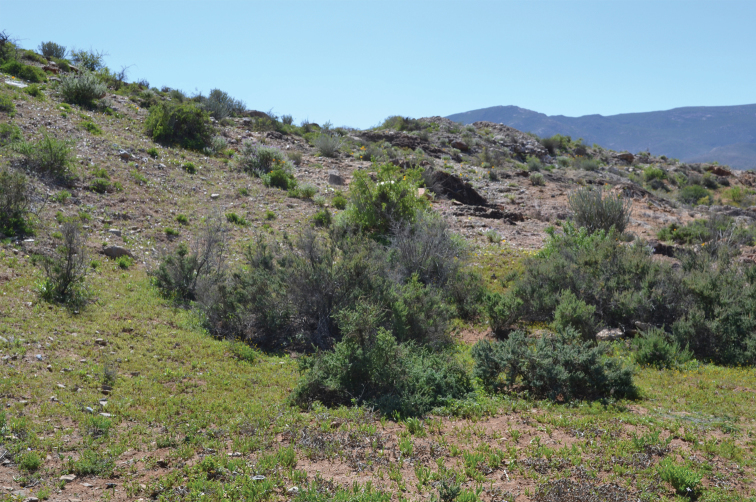
Type locality of *Kuboesphasma compactum* sp. n., Kuboes, Richtersveld, South Africa.

**Table 1.** Overview of the type material including gender, locality, collection date and museum where it is deposited. Abbreviations: SAMC – Iziko South African Museum, Cape Town, South Africa. ZFMK – Zoologisches Forschungsinstitut und Museum Alexander Koenig, Bonn, Germany; ZMBN – University Museum, University of Bergen, Bergen, Norway.

	Type	Gender	Locality	Collection date	Deposition
*Kuboesphasma compactum*	Holotype	♂	S28°26'25", E16°59'44"	18.viii.2012	SAMC
	Paratype	♂	S28°26'25", E16°59'44"	18.viii.2012	SAMC
	Paratype	♂	S28°26'25", E16°59'44"	18.viii.2012	ZFMK
	Paratype	♂	S28°26'25", E16°59'44"	18.viii.2012	ZFMK
	Paratype	♂	S28°26'25", E16°59'44"	18.viii.2012	ZMBN
	Paratype	♀	S28°26'25", E16°59'44"	18.viii.2012	SAMC
	Paratype	♀	S28°26'25", E16°59'44"	18.viii.2012	ZMBN
*Minutophasma richtersveldense*	Holotype	♂	S28°47'26.94", E17°16'20.34"	06.ix.2014	SAMC
	Paratype	♂	S28°47'26.94", E17°16'20.34"	06.ix.2014	SAMC
	Paratype	♂	S28°47'26.94", E17°16'20.34"	06.ix.2014	ZMBN
	Paratype	♂	S28°47'26.94", E17°16'20.34"	06.ix.2014	ZMBN
	Paratype	♂	S28°47'26.94", E17°16'20.34"	06.ix.2014	ZFMK
	Paratype	♀	S28°47'26.94", E17°16'20.34"	06.ix.2014	SAMC
	Paratype	♀	S28°47'26.94", E17°16'20.34"	06.ix.2014	ZMBN
	Paratype	♀	S28°47'26.94", E17°16'20.34"	06.ix.2014	ZMBN
	Paratype	♀	S28°47'26.94", E17°16'20.34"	06.ix.2014	ZFMK
	Paratype	♂	S28°46'31.50", E17°11'12.18"	06.ix.2014	SAMC
	Paratype	♂	S28°46'31.50", E17°11'12.18"	06.ix.2014	ZMBN
	Paratype	♀	S28°46'31.50", E17°11'12.18"	06.ix.2014	SAMC
	Paratype	♀	S28°46'31.50", E17°11'12.18"	06.ix.2014	ZMBN
	Paratype	♀	S28°10'20.80", E17°01'43.60"	07.ix.2014	ZMBN
	Paratype	♀	S28°10'20.80", E17°01'43.60"	07.ix.2014	SAMC

#### Description male.

Measurements (male holotype followed by paratypes in parentheses, in mm): total length: 12.1 (12.5, 12.9, 12.1, 12.4); length of pronotum: 2.5 (2.6, 2.8, 2.6, 2.8); width of pronotum: 2.5 (2.4, 2.6, 2.3, 2.4); length of mesonotum: 2.0 (2.2, 2.1, 2.1, 2.3); width of mesonotum: 2.4 (2.3, 2.4, 2.2, 2.3); length of metanotum: 1.5 (1.9, 1.7, 1.7, 1.7); width of metanotum: 2.2 (2.2, 2.2, 2.1, 2.1); heights of head: 2.3 (2.3, 2.2, 2.2, 2.3); total heights of head: 2.9 (3.0, 2.9, 2.9, 3.0); width of the head: 2.8 (2.9, 3.0, 2.7, 2.9); head width over eyes: 3.0 (3.0, 3.2, 3.0, 3.0); width between eyes: 1.7 (1.7, 1.8, 1.7, 1.6); length of eye: 1.3 (1.2, 1.4, 1.4, 1.4); width of eye: 0.8 (0.8, 0.9, 0.8, 0.8).

**Figure 4.**
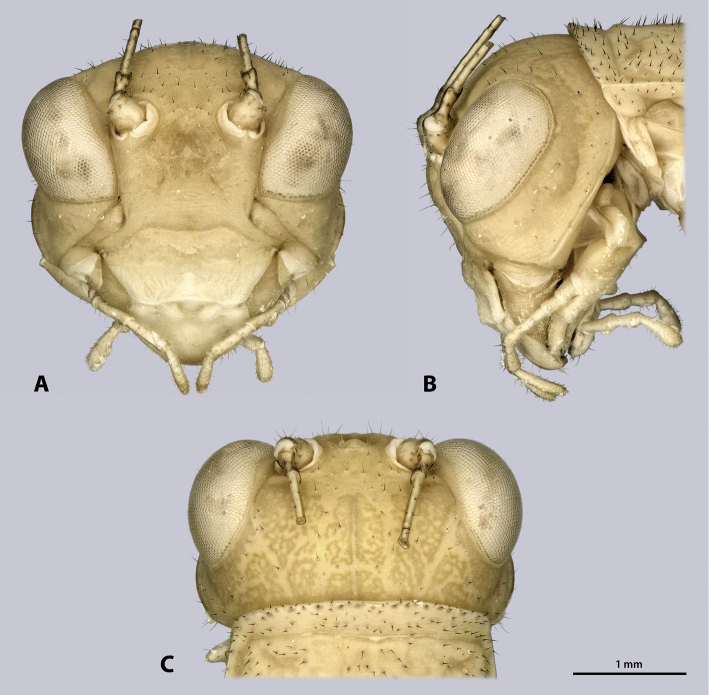
Head of ♂ *Kuboesphasma compactum* sp. n., holotype, photomicrographies **A** frontal view **B** lateral view **C** dorsal view.

Head (Fig. 4): globular, orthognathous, posteriorly covered by pronotum, green without darker stripe; compound eyes whitish with black or brown spots; head slightly wider than prothorax, about twice as wide as long; head capsule covered with setae, setation increasing dorsally. Compound eyes prominent, tapered ventro-mesally, about 1.5 times as long as high; interoccular distance ca. the length of one eye, ocelli absent. Coronal and frontal suture indistinct, pleurostomal ridge well developed. Ventral parts of occipital ridge very prominent; antennal sockets in between eyes, distinct; interantennal distance ca. diameter of one antennal socket; antennifer present; dark spot on lateral corner of scapus absent; dark median butterfly-shaped spot directly below the antennal bases washed-out and indistinct; anterior tentorial pits dorso-mesally of anterior mandibular articulation; frons with three bulges, one in between antennal sockets, two ventro-mesal of antennal sockets; frontoclypeal ridge recognizable as an indistinct line. Gena strongly protruding, head capsule on the level of the genae nearly as wide as on the one of the compound eyes; genae have equal heights than the compound eyes. Clypeus trapezoid, with well-developed clypeolabral ridge, no setae present; oval sclerite in between clypeus and labrum present. Labrum flat, anteriorly rounded, with few short setae. Maxilla well developed, green; maxillary palp five segmented, sparsely covered with setae, palpomere one and two as long as wide, palpomere three 2.5 times as long as wide, palpomeres four and five ca. twice as long as wide. Labium green, palp three segmented. Scape and pedicel bright green; scape as long as wide; pedicle half as wide as scape, twice as long as wide, dilating towards the tip. Flagellum slightly shorter than the entire animal.

**Figure 5.**
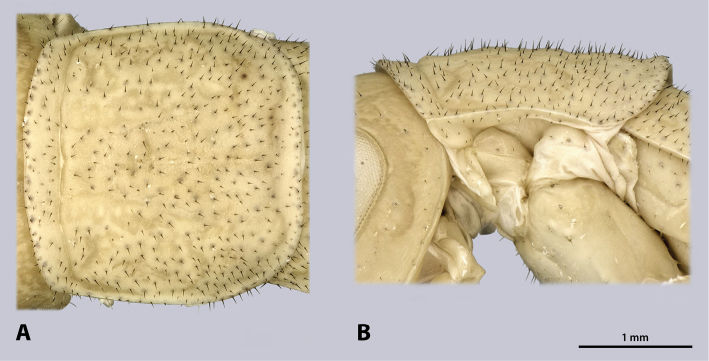
Prothorax of ♂ *Kuboesphasma compactum* sp. n., holotype, photomicrographies; **A** dorsal view **B** lateral view.

Thorax (Fig. 5): bright green, dorso-medially with weak and indistinct longitudinal brown stripe that contains small green areas. Notae covered with setae, much denser than head. Pronotum oval with bulge positioned anterior-laterally; pronotum reaches overhead and mesonotum, ventral border of pronotum arched. Two cervicalia present, second postero-dorsally to first. Pleura subdivided into epimeron and episternum. Coxae large, covered with setae.

Legs: green, spikes in the tibial region black; covered with setae. Prothoracic leg more massive than meso- and metathoracic ones; femur ca. three times as long as wide, with two ventro-median rows of spikes, spikes larger on pro- and mesothoracic leg, smaller on metathoracic one. Tibia green, in pro- and mesothorax ca. 10 times as long as wide, in metathoracic leg between 13 and 15 times as long, with two ventro-median rows of black spikes on pro- and mesothoracic legs, protibia with 9–12 spikes per row, on metathoracic leg only two distal spikes. Tarsus with five tarsomeres, proximal four tarsomeres with euplantulae; arolium very large.

Wings: completely absent.

Abdomen: as long as thorax and head combined; green, meso-dorsal brown longitudinal stripe weak and indistinct. Abdomen covered with setae, tergites stronger than sternits. Abdominal tergum I same width or very slightly thinner as metathorax; terga slightly broadening towards tergum VIII, terga IX and X narrowing again.

**Figure 6.**
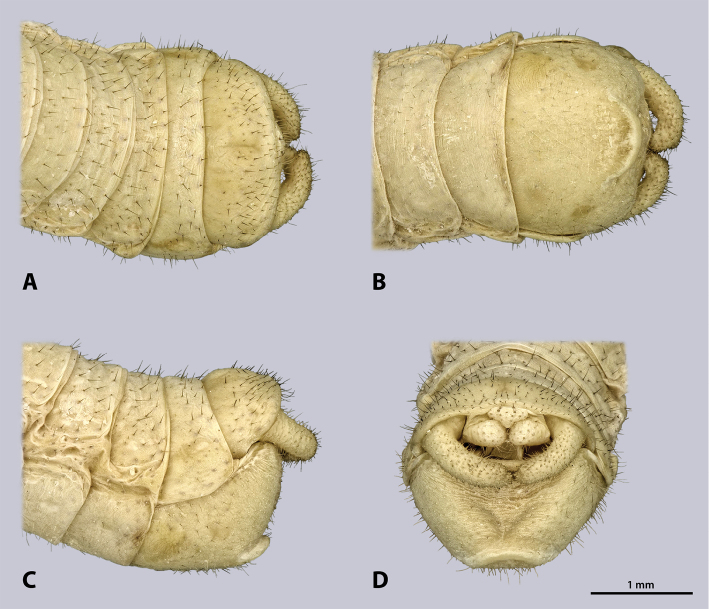
Terminalia of ♂ *Kuboesphasma compactum* sp. n., holotype, photomicrographies; **A** dorsal view **B** ventral view **C** lateral view **D** caudal view.

Male terminalia (Fig. 6): tergum IX green; shorter than tergum VIII. Tergum X green, mesal brown stripe, roof-shaped in lateral view. Subgenital plate (sternite IX) green with brownish areas, posterio-dorsal margin not protruding; process of subgenital plate broad, dorsal slightly arch-shaped when seen from posterior, broadly emarginated dorsally. Cerci one-segmented, densely covered with setae; diameter mesally round, uniformly curved, slightly narrowed towards the apex, with distal dorsal projection; cerci extending towards the middle of the subgenital plate. Paraprocts and epiproct also covered with setae.

#### Description female.

For the female only differences to the male are described. Measurements: total length: 13.5, 15.4; length of pronotum: 3.2, 3.4; width of pronotum: 3.2, 3.7; length of mesonotum: 2.0, 2.5; width of mesonotum: 3.0, 3.5; length of metanotum: 1.5, 2.0; width of metanotum: 2.8, 3.4; heights of head: 2.5, 2.8; total heights of the head: 3.2, 3.9; width of the head: 3.2, 3.8; head width over eyes: 3.4, 3.8; width between eyes: 2.1, 2.3; length of eye: 1.5, 1.7; width of eye: 0.8, 0.9.

**Figure 7.**
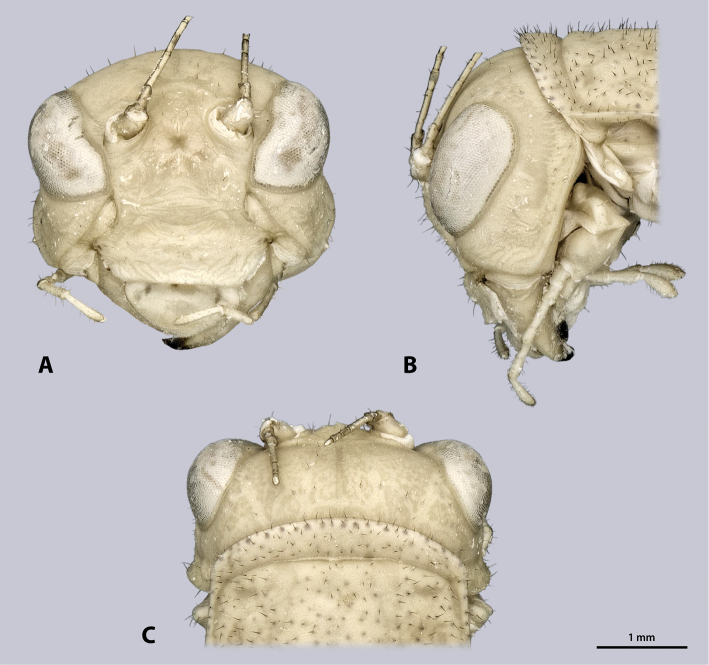
Head of ♀ *Kuboesphasma compactum* sp. n., paratype, photomicrographies; **A** frontal view **B** lateral view **C** dorsal view.

Head (Fig. 7): compound eyes slightly smaller than in the male.

**Figure 8.**
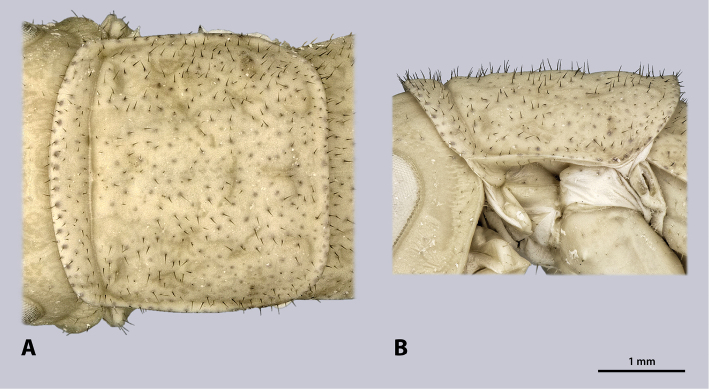
Prothorax of ♀ *Kuboesphasma compactum* sp. n., paratype, photomicrographies; **A** dorsal view **B** lateral view.

Thorax (Fig. 8): dorsal stripe absent. Ventral border of pronotum straight.

Legs: protibia with 9–10 spikes per row.

Abdomen: abdomen slightly longer than head and thorax combined. No dorsal brown stripe. Widest point of abdomen at segments 5 or 6.

**Figure 9.**
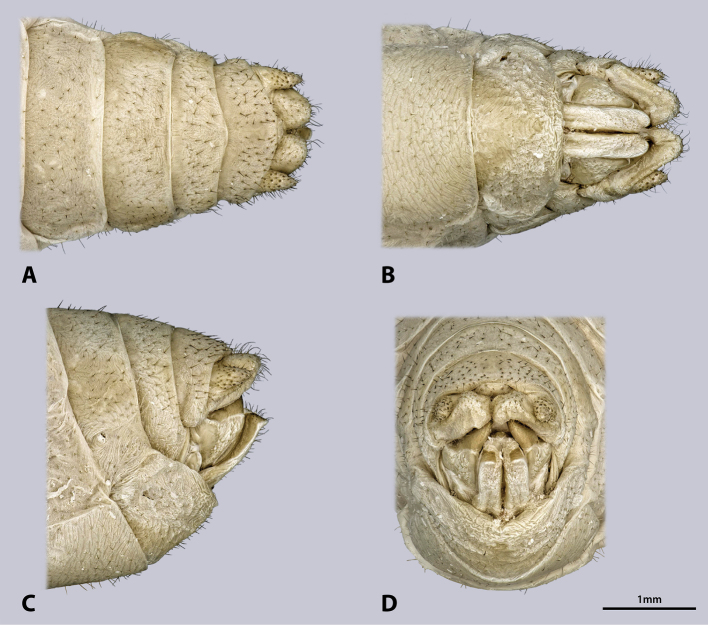
Terminalia of ♀ *Kuboesphasma compactum* sp. n., paratype, photomicrographies; **A** dorsal view **B** ventral view **C** lateral view **D** caudal view.

Female terminalia (Fig. 9): tergum IX green, approximately as long as tergum VIII, posterior margin with distinct median convexity. Tergum X green, as long as tergum IX; apex rounded posteriorly; all terga setose; epiproct green, very short (ca. 1/5 of the length of tergum X), slightly setose. Paraprocts rounded and densely covered with setae. Cerci slightly shorter than paraprocts, cone shaped and densely covered with setae. Sternite VIII green, posterior margin straight. Gonapophysis VIII long and slender, distally blunt with ventrocaudal process. Gonocoxite IX almost completely concealed in lateral view; gonoplac triangular, heavily sclerotized.

#### Etymology.

The species name *compactum* refers to the compact appearance of that species which distinguishes it easily from the second greenish species in the Richtersveld, *Minutophasma richtersveldense*.

#### Comments.

Specimens were common in a heavily overgrazed area near the settlement of Kuboes; mainly in dense shrubs with small green and succulent leaves (*Suaeda fruticosa*, *Lycium* sp.).

### Minutophasma gen. n.

Taxon classificationAnimaliaNotopteraMantophasmatidae

http://zoobank.org/24A52AD5-F6E8-4BA3-B5BF-38E069F1EC59

#### Description and diagnosis.

*Minutophasma* gen. n. is placed as sistergroup to the Namibian genus *Striatophasma*.

*Minutophasma* can be distinguished from other mantophasmatodeans except *Kuboesphasma* by the washed-out and indistinct butterfly-shaped spot on the frons: in the South African Austrophasmatidae sensu Klass et al. (2003) except *Viridiphasma clanwilliamense* this spot is clear and dark while *V. clanwilliamense* and the Namibian *Striatophasma*, *Pachyphasma* and Mantophasmatidae sensu Klass et al. (2003) lack it completely. Additionally, it can be distinguished from all Austrophasmatidae sensu Klass et al. (2003) except *Viridiphasma* by genae that are similar long in lateral view as the widths of the eyes. *Kuboesphasma* differs from *Minutophasma* by the larger body size (males over 12 mm and females over 13 mm), an indistinct dark dorsal stripe in males and the absence of ventro-lateral spot on the scapus. On the protibia, the males of *Kuboesphasma* have nine or more spikes per row (while *Minutophasma* has eight or less). Additionally, the head capsule is distinctly broader on the level of the compound eyes than on the level of the genae in *Minutophasma* while they are equal in *Kuboesphasma*. Males and females of *Praedatophasma maraisi* are much larger (more than 21 mm) than those of *Kuboesphasma*, grey brown and have distinct spines along the thorax. In contrast to the reddish-brown *Namaquaphasma ookiepense*, specimens of *Minutophasma* are mostly green, much smaller (males of *Namaquaphasma* above 14 mm and females above 16 mm) and have genae that are lower than the eyes are wide. Specimens of *Striatophasma naukluftense* are larger (males 12–15 mm, females 16–27 mm) as those of *Minutophasma* and lack the dorsal distal projection in male cerci. All green species of Austrophasmatidae (*Austrophasma gansbaaiense*, *A. caledonense*, *Viridiphasma*, *Lobatophasma*) and *Striatophasma* lack the ventrolateral black spot on the scapus which is very distinct in *Minutophasma*.

#### Type species.

*Minutophasma richtersveldense*

#### Other included species.

None thus far.

#### Etymology.

The generic group name *Minutophasma* is a composition of the Latin word minutus that refers to the small size of that species which separates it from the other known mantophasmatodeans, and the ending -phasma which is commonly used to term mantophasmatodeans. The gender is neuter.

### Minutophasma richtersveldense sp. n.

Taxon classificationAnimaliaNotopteraMantophasmatidae

http://zoobank.org/D4CD7E84-1243-4F1E-87C6-D211A05B3D6B

Figs 10 , 11 , 12 , 13 , 14 , 15 , 16 , 17

#### Holotype.

Male. SOUTH AFRICA: Northern Cape, north of Eksteenfontein, Richtersveld, S28°47'26.94", E17°16'20.34", 06.ix.2014, 600–700m, R. Predel, specimen in ethanol. Table 1 provides an overview of the type material including the collections where it is deposited.

#### Paratypes.

Location 1: SOUTH AFRICA, Northern Cape, north of Eksteenfontein, Richtersveld, S28°47'26.94", E17°16'20.34", 06.ix.2014, 600–700m, R. Predel: 4 males and 4 females, specimens in ethanol. Location 2: SOUTH AFRICA, Northern Cape, west of Eksteenfontein, Richtersveld, S28°46'31.50", E17°11'12.18", 06.ix.2014, 500m, R. Predel: 2 males and 2 females, specimens in ethanol. Location 3: SOUTH AFRICA, Northern Cape, Akkedis pass, Richtersveld, S28°10'20.80", E17°01'43.60", 07.ix.2014, R. Predel: 2 females, specimens in ethanol. Table 1 provides an overview of the type material including the collections where it is deposited.

#### Description male.

Measurements (male holotype followed by paratypes in parentheses, in mm): total length: 9.2 (location 1: 9.1, 10.5, 9.8, 9.9, 10.7) (location 2: 10.6, 10.1); length of pronotum: 1.8 (location 1: 1.9, 1.9, 1.6, 1.9, 1.6) (location 2: 2.0, 2.1); width of pronotum: 1.6 (location 1: 1.7, 1.8, 1.5, 1.8, 1,6) (location 2: 1.9, 1.8); length of mesonotum: 1.7 (location 1: 1.7, 1.7, 1.5, 1.8, 1.5) (location 2: 1.8, 1.8); width of mesonotum: 1.5 (location 1: 1.6, 1.6, 1.4, 1.7, 1.5) (location 2: 1.7, 1.6); length of metanotum: 1.3 (location 1: 1.3, 1.4, 1.4, 1.4, 1.2) (location 2: 1.4, 1.3); width of metanotum: 1.3 (location 1: 1.4, 1.5, 1.3, 1.5, 1.5) (location 2: 1.4, 1.4); heights of head: 1.7 (location 1: 1.6, 1.7, 1.5, 1.6, 1.5) (location 2: 1.7, 1.6); total heights of head: 2.1(location 1: 2.1, 2.1, 1.9, 2.0, 1.9) (location 2: 2.2, 2.2); width of the head: 2.0 (location 1: 2.0, 2.0, 1.8, 2.2, 1.8) (location 2: 2.3, 2.2); head width over eyes: 2.2 (location 1: 2.3, 2.3, 2.1, 2.4, 2.1) (location 2: 2.4, 2.4); width between eyes: 1.3 (location 1: 1.3, 1.4, 1.3, 1.3, 1.2) (location 2: 1.5, 1.5); length of eye: 1.0 (location 1: 1.0, 1.0, 1.0, 1.0, 1.1) (location 2: 1.0, 1.1); width of eye: 0.6 (location 1: 0.6, 0.6, 0.6, 0.6, 0.6) (location 2: 0.6, 0.6).

**Figure 10.**
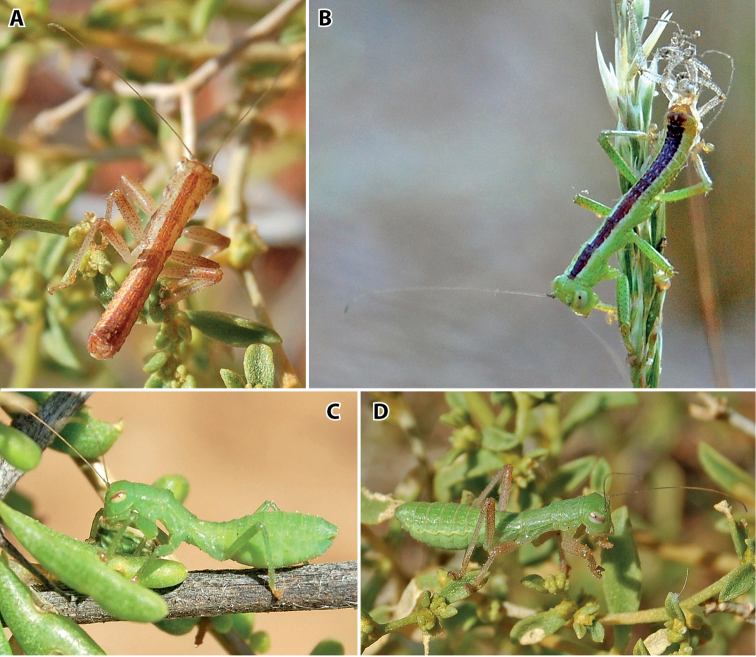
Habitus photographies and color variations of *Minutophasma richtersveldense* sp. n.; **A** ♂ with brown body color **B** ♂ with green body color **C** ♀ with green body color **D** ♀ with green body color and brown legs.

Coloration (Fig. 10): body color ranges from green to brown to grey-beige (Fig. 10). Distinct and broad dark stripe on dorsal side, stripe in green males with whitish margins.

Head (Fig. 12): globular, orthognathous, posteriorly covered by pronotum, in some specimens the dark stripe is indistinctly visible on the vertex but weaker than on the thorax and abdomen; head slightly wider than prothorax, about twice as wide as long; head capsule sparsely covered with setae. Compound eyes whitish with black or brown spots, prominent, tapered ventro-mesally, about 1.5 times as long as high; interoccular distance ca. the length of one eye, ocelli absent. Coronal and frontal suture indistinct, pleurostomal ridge well developed. Ventral parts of occipital ridge very prominent; antennal sockets in between eyes, distinct; interantennal distance ca. diameter of one antennal socket; antennifer present; dark median butterfly-shaped spot directly below the antennal bases present but washed out and indistinct, size and pigmentation varying between specimens; anterior tentorial pits dorso-mesally of anterior mandibular articulation; frons with three bulges, one in between antennal sockets, two ventro-mesal of antennal sockets; frontoclypeal ridge not recognizable. Gena not strongly protruding, head capsule on the level of the genae distinctly narrower than on the one of the compound eyes; heights of genae lower than heights of compound eyes. Clypeus trapezoid, with well-developed clypeolabral ridge, oval sclerite in between clypeus and labrum present. Labrum flat, anteriorly rounded, with few short setae. Maxilla well developed; maxillary palp five segmented, sparsely covered with setae, palpomere one and two as long as wide, palpomere three 2.5 times as long as wide, palpomeres four and five ca. twice as long as wide. Labial palp three segmented. Scape as long as wide, with distinct black ventro-lateral spot; pedicle half as wide as scape, twice as long as wide, dilating towards the tip. Flagellum about as long as the entire animal.

**Figure 11.**
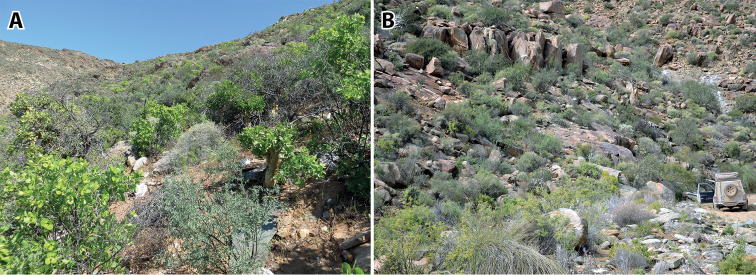
Type locality of *Minutophasma richtersveldense* sp. n., Eksteenfontein, Richtersveld, South Africa.

**Figure 12.**
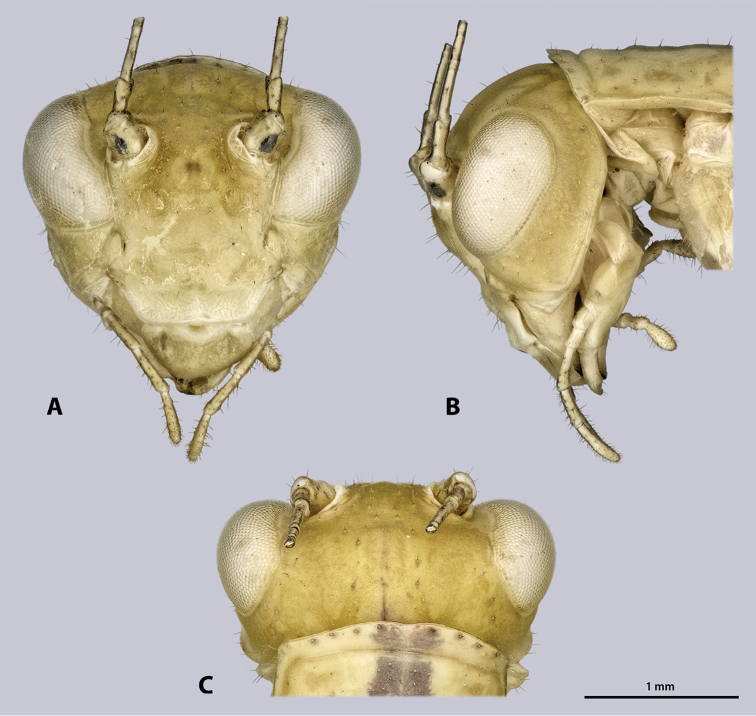
Head of ♂ *Minutophasma richtersveldense* sp. n., holotype, photomicrographies; **A** frontal view **B** lateral view **C** dorsal view.

**Figure 13.**
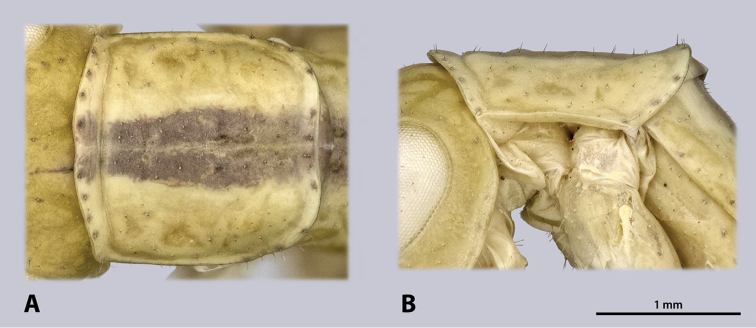
Prothorax of ♂ *Minutophasma richtersveldense* sp. n., holotype, photomicrographies; **A** dorsal view **B** lateral view.

Thorax (Fig. 13): dorso-medially with distinct and broad longitudinal dark stripe. Notae sparsely covered with setae. Pronotum oval with bulge positioned anterior-laterally; pronotum reaches over head and mesonotum, ventral boarder of pronotum straight. Two cervicalia present, second located postero-dorsally to the first. Pleura subdivided into epimeron and episternum. Coxae large, covered with setae.

Legs: tibia with black spikes, covered with setae. Prothoracic leg more massive than meso- and metathoracic ones; profemur ca. 4 times, mesofemur ca. 4–5 times and metafemur 6–8 times as long as wide, all legs with two ventro-median rows of spikes, spikes in some specimens larger on pro- and mesothacic leg, smaller on metathoracic one. Tibia, in pro- and mesothorax ca. 8–11 times as long as wide, in metathoracic leg between 14 and 16 times as long, with two ventro-median rows of black spikes on pro- and mesothoracic legs, protibia with 5–8 spikes per row, on metathoracic leg only two distal spikes. Tarsus with five tarsomeres, proximal four tarsomeres with euplantulae; arolium very large.

Wings: completely absent.

Abdomen: as long as thorax and head combined; meso-dorsal dark longitudinal stripe strongly developed. Abdomen covered with setae. Abdominal tergum I same width or very slightly thinner as metathorax; terga slightly broadening towards tergum IX, tergum X narrowing again.

**Figure 14.**
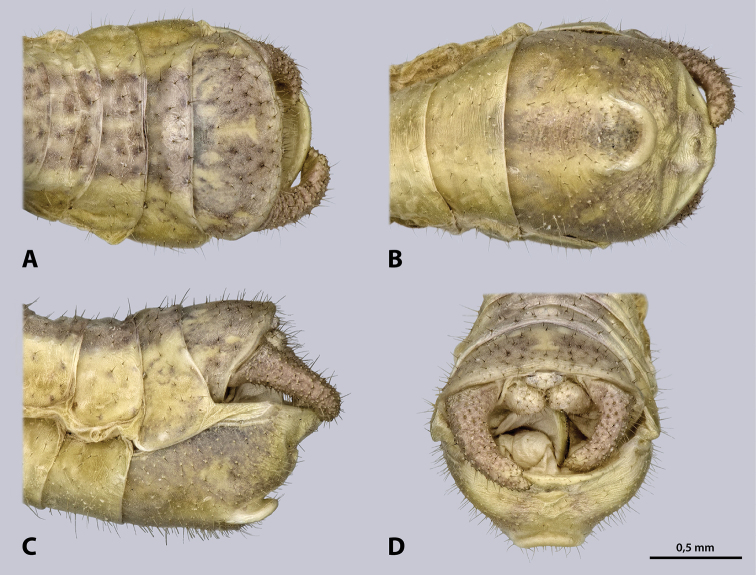
Terminalia of ♂ *Minutophasma richtersveldense* sp. n., holotype, photomicrographies; **A** dorsal view **B** ventral view **C** lateral view **D** caudal view.

Male terminalia (Fig. 14): tergum IX shorter than tergum VIII, posterior margin concave. Dark stripe on tergum X much broader than on previous segments, almost covering entire tergum, roof-shaped in lateral view. Subgenital plate (sternite IX) large, with strongly protruding posterio-dorsal margin; process of subgenital plate broad, almost straight when seen from posterior, broadly emarginated dorsally. Cerci one segmented, densely covered with setae; diameter round, uniformly curved, narrowed towards the apex; cerci with dorsal projection and extending towards the middle of the subgenital plate. Paraprocts and epiproct also covered with setae.

#### Description female.

For the female only differences to the male are described. Measurements: total length: (location 1: 11.3, 12.6, 12.7, 10.7) (location 2: 11.2, 13.2) (location 3: 12.3, 12.5); length of pronotum: (location 1: 2.2, 2.4, 2.3, 2.0) (location 2: 2.2, 2.1) (location 3: 2.2, 2.3); width of pronotum: (location 1: 2.0, 2.3, 2.2, 1.7) (location 2: 2.1, 2.0) (location 3: 2.0, 2.2); length of mesonotum: (location 1: 1.9, 2.0, 2.0, 1.8) (location 2: 2.0, 1.9) (location 3: 2.0, 1.9); width of mesonotum: (location 1: 1.9, 2.1, 2.0, 1.6) (location 2: 1.9, 1.8) (location 3: 1.8, 2.1); length of metanotum: (location 1: 1.4, 1.6, 1.6, 1.3) (location 2: 1.3, 1.5) (location 3: 1.5, 1.4); width of metanotum: (location 1: 1.8, 2.0, 1.8, 1.6) (location 2: 1.8, 1.8) (location 3: 1.7, 2.0); heights of head: (location 1: 1.6, 2.1, 2.1, 1.8) (location 2: 1.9, 2.1) (location 3: 1.8, 2.1); total heights of the head: (location 1: 2.4, 2.7, 2.7, 2.4) (location 2: 2.6, 2.7) (location 3: 2.4, 2.7); width of the head: (location 1: 2.4, 2.5, 2.4, 2.2) (location 2: 2.4, 2.4) (location 3: 2.3, 2.4); head width over eyes: (location 1: 2.6, 2.7, 2.8, 2.4) (location 2: 2.7, 2.7) (location 3: 2.5, 2.5); width between eyes: (location 1: 1.6, 1.7, 1.6, 1.4) (location 2: 1.7, 1.7) (location 3: 1.7, 1.7); length of eye: (location 1: 1.1, 1.2, 1.1, 1.0) (location 2: 1.2, 1.1) (location 3: 1.1, 1.2); width of eye: (location 1: 0.6, 0.7, 0.6, 0.6) (location 2: 0.7, 0.6) (location 3: 0.7, 0.7).

Coloration: all found females are green, without dorsal longitudinal dark stripe.

**Figure 15.**
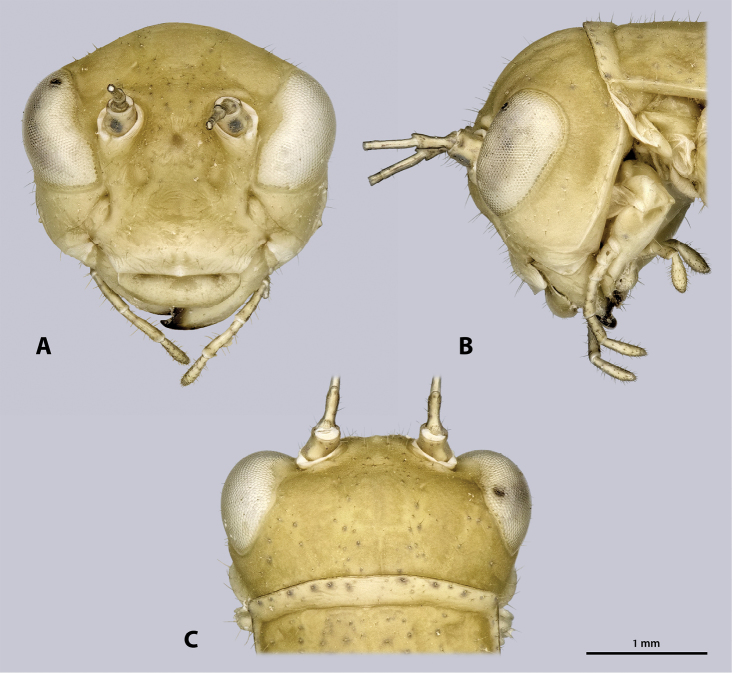
Head of ♀ *Minutophasma richtersveldense* sp. n., paratype, photomicrographies; **A** frontal view **B** lateral view **C** dorsal view.

Head (Fig. 15): compound eyes slightly smaller than in the male. Head capsule on the level of the genae distinctly wider than on the level of the compound eyes.

**Figure 16.**
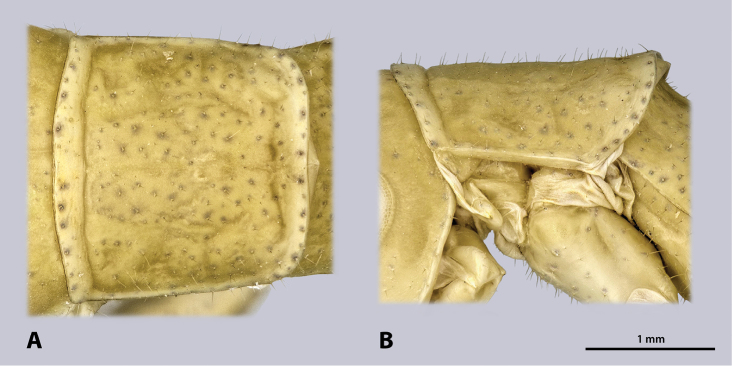
Prothorax of ♀ *Minutophasma richtersveldense* sp. n., paratype, photomicrographies **A** dorsal view **B** lateral view.

Thorax (Fig. 16): notae with slightly denser setation than males. No dorsal dark stripe.

Legs: protibia with 6–9 spikes per row.

Abdomen: no dorsal brown stripe. Widest point of abdomen at segments 5 or 6.

**Figure 17.**
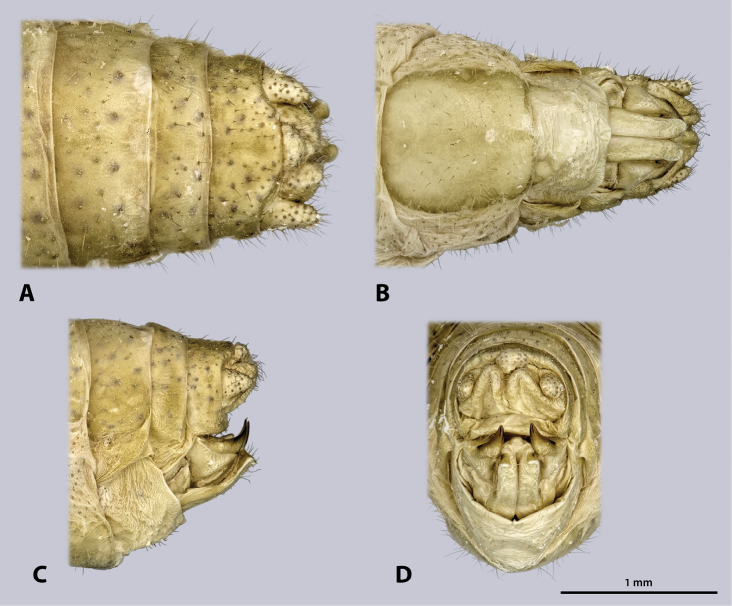
Terminalia of ♀ *Minutophasma richtersveldense* sp. n., paratype, photomicrographies; **A** dorsal view **B** ventral view **C** lateral view **D** caudal view.

Female terminalia (Fig. 17): tergum IX shorter than tergum VIII, posterior margin without distinct convexity. Tergum X slightly longer as tergum IX; apex rounded posteriorly; terga with sparse setation; epiproct half as long as tergum X, setose. Paraprocts rounded and densely covered with setae. Cerci slightly shorter than paraprocts, cone shaped and densely covered with setae. Sternite VIII with straight posterior margin. Gonapophysis VIII long and slender, distally blunt with ventrocaudal process. Gonocoxite IX almost completely hidden in lateral view; gonoplac triangular, heavily sclerotized.

#### Etymology.

The species name *richtersveldense* refers to the currently known area of distribution, the Richtersveld.

#### Comments.

This species was found in a variety of green and grey-green bushes with small leaves; several specimens were also collected from grass stalks. From the most northern population at Akkedis pass, only females or female nymphs (>20) were recorded over a period of three years. The absence of males implies that parthenogenesis might occur; this phenomenon was not reported from mantophasmatodeans so far. The sex ratio in the populations around Eksteenfontein was about 1:1 as usual in Mantophasmatodea.

## Discussion

**Figure 18.**
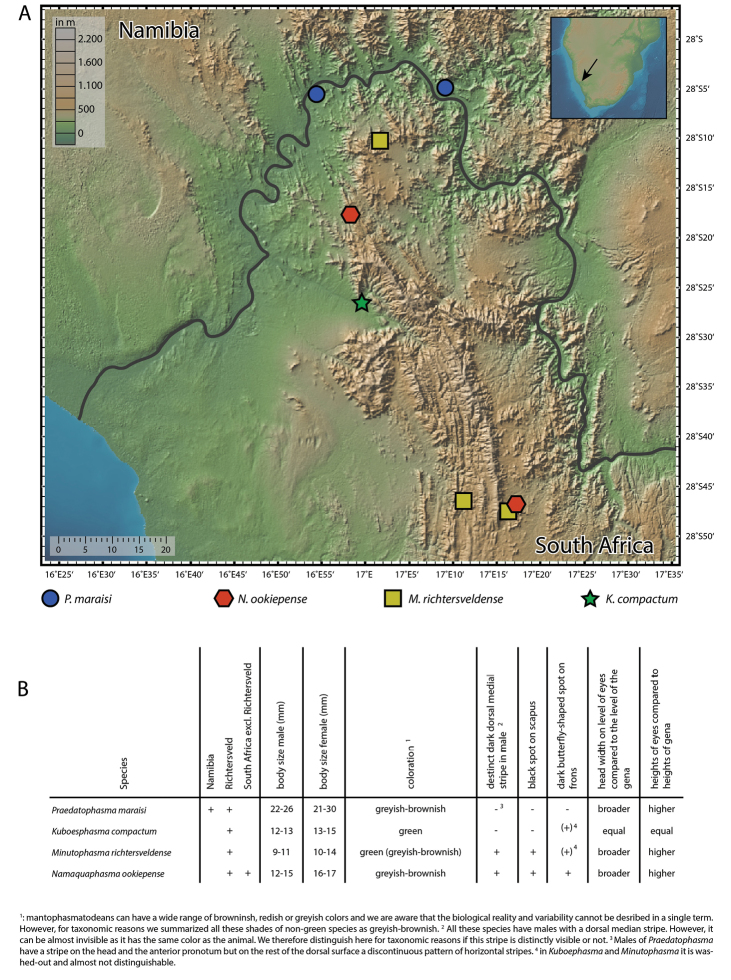
**A** map of the Richtersveld with the reported findings of the four mantophasmatodean species (scale in km) **B** table comprising the most important features to distinguish the four species of Mantophasmatodea in the Richtersveld.

The Richtersveld region is part of the Great Escarpment and an exceptional center of endemism within the Succulent Karoo (Hilton-Taylor 1996). A characteristic feature of the Richtersveld is its location in the transitional area between the summer rain (found north-east of the Richtersveld) and the south-western winter rain region in South Africa, which is accompanied by diverse topographic conditions. Apparently, these circumstances promoted the occurrence of locally distributed taxa and hence, speciation which is also observed in Mantophasmatodea. More precisely, the Richtersveld region is inhabited by not less than four easily distinguishable mantophasmatodean species. Figure 18 provides a table with important taxonomic features to separate these species. Due to a sporadic appearance of these insects, caused by irregular rainfall patterns and the complex topographic features in the Richtersveld, there is only preliminary information about the distribution of these taxa. Even though the data provided in this manuscript result from four field excursions into this comparably small area, *Kuboesphasma compactum* sp. n. was only found in a heavily overgrazed habitat (ca. 2 km^2^) near the village of Kuboes (Fig. 18) and therefore seems to have a very limited distribution. This species, which likely represents the sister group of all South African Austrophasmatidae sensu Klass et al. (2003) (Predel, unpublished data), was not yet observed in neighboring areas with less disturbed vegetation. In contrast, *Minutophasma richtersveldense* sp. n. is reported from southern and northern localities in the Richtersveld (see Fig. 18), which confirms a wider distribution in that region. In addition to these two novel taxa, which are endemic to the Richtersveld, members of two other lineages enter this region and reach their currently known distribution boundaries in the Richtersveld. The large *Praedatophasma maraisi* seems to have its southern boundary of distribution just south of the Orange River. This species was described from a single female found at the Namibian-South African border at the mouth of the Fish River into the Orange River (Zompro et al. 2002; Fig. 18). We found another single female and a single male nymph in a very dry and sandy area near Sendelingsdrif at the northern boundary of the Richtersveld in South Africa (Fig. 18), but could not find any further specimens again. However, another population of this species was reported from the very dry Gaab/Fish River region approximately 90 km north-west of the above-mentioned localities (EduVentures Namibia; see Roth et al. 2014). Different from the other species in the Richtersveld, *P. maraisi* is obviously restricted to the vicinity of mostly dried-out riverbeds and its coloration suggests a ground-orientated lifestyle. The fourth species found in the Richtersveld is *Namaquaphasma ookiepense* (Klass et al. 2003). It is a common and widely distributed species in the Northern Cape province of South Africa. The Richtersveld, where this species seems to be rather uncommon, constitutes the northernmost extension of its distribution. Around Eksteenfontein *N. ookiepense* was found in sympatry with *M. richtersveldense*, but size and coloration facilitate a clear distinction of these species in the field (Fig. 18). Towards the south, this species is found to an assumed line from Strandfontein inland to Nuwerus about 400 km south of the Richtersveld. There it is replaced by various other species of Austrophasmatidae which are common in the south-westernmost regions of the Northern Cape and the Western Cape (see Roth et al. 2014).

The comparatively high diversity of Mantophasmatodea in the Richtersveld stands in strong contrast to other regions within their currently known distribution, where even sympatry of two species is rare (Eberhard et al. 2011; Predel et al. 2012). This diversity could be interpreted as an indicator of speciation in this area. Yet, the phylogenetic positions of these taxa (Predel, unpublished data; Fig. 1) rather suggest, that the Richtersveld was colonized multiple times independently. *Praedatophasma maraisi*, together with the closely related *Tyrannophasma gladiator* from the Brandberg in northern Namibia, constitute the sistergroup of the remaining mantophasmatids. It therefore probably colonized the Richtersveld independently from the other species in that region. The South African taxa of Mantophasmatodea, including *Kuboesphasma* and *Minutophasma* from the Richtersveld, form a monophyletic clade with the southern Namibian *Striatophasma* (Fig. 1). The Richtersveld thus provided an important gateway in the dispersal of Mantophasmatodea from the summer-rain regions of southern Namibia towards the winter-rain areas of South Africa, where a distinct speciation took place (Klass et al. 2003; Predel et al. 2012; Roth et al. 2014).

## Supplementary Material

XML Treatment for Kuboesphasma

XML Treatment for Kuboesphasma compactum

XML Treatment for Minutophasma

XML Treatment for Minutophasma richtersveldense
